# Interaction between Neuronal Depolarization and MK-801 in SH-SY5Y Cells and the Rat Cortex

**DOI:** 10.4306/pi.2008.5.2.94

**Published:** 2008-06-30

**Authors:** Yeni Kim, Miran Seo, Yun-Il Lee, So-Young Kim, Eun-Ah Cho, Se-Hyun Kim, Yong-Min Ahn, Ung-Gu Kang, Yong-Sik Kim, Yong-Sung Juhnn

**Affiliations:** 1 Department of Biochemistry and Molecular Biology, Seoul National University College of Medicine, Seoul, Korea.; 2 Department of Psychiatry and Behavioral Science and Institute of Human Behavioral Medicine, Seoul National University College of Medicine, Seoul, Korea.

**Keywords:** AKT, Glycogen synthase kinase 3β, Extracellular legulated kinase 1/2, KCl, Electro convulsive shock, MK-801

## Abstract

**Objective:**

The interaction between MK-801, a model of psychosis and KCl-induced depolarization or electroconvulsive shock (ECS), a therapeutic model of electroconvulsive therapy (ECT), was investigated in SH-SY5Y cells and the rat frontal cortex.

**Methods:**

SH-SY5Y cells were pretreated with 1 µM MK-801 for 15 min, followed by cotreatment with 100 mM KCl for 5 min. MK-801 was reintroduced after the KCl was washed out, and the samples were incubated before harvesting. For the experiments in rats, male Sprague-Dawley rats were treated with MK-801 followed by ECS. Immunoblot analyses of glycogen synthase kinase 3β (GSK3β) (Ser9), AKT (Ser473) and extracellular legulated kinase (ERK)1/2 in SH-SY5Y cells and the rat frontal cortex were performed.

**Results:**

KCl-induced neuronal depolarization resulted in the transient dephosphorylation of AKT (Ser473) and GSK3β (Ser9), followed by increased phosphorylation of the enzymes in SH-SY5Y cells. Cotreatment with MK-801 and KCl inhibited the initial dephosphorylation of AKT and GSK3β produced by KCl-induced neuronal depolarization. Similarly, ECS resulted in the transient dephosphorylation of AKT (Ser473) and GSK3β (Ser9), whereas cotreatment with MK-801 inhibited the initial dephosphorylation of AKT (Ser473) and GSK3β (Ser9) produced by ECS in the rat frontal cortex. No significant interaction was observed between MK-801 and KCl in the dephosphorylation of ERK1/2.

**Conclusion:**

These results suggest that an antagonistic interplay between MK-801 and neuronal depolarization by KCl or ECS is involved the regulation of AKT (Ser473) and GSK3β (Ser9) phosphorylation.

## Introduction

Intracellular signal interference may play a substantial role in the development of neuropathological processes in psychosis, thus the elimination of the cause of the interference may be a potential target in the treatment of psychosis. Complex arrays of intracellular signal transduction pathways lead to changes in cellular function. To determine the relationship between pathological processes and recovery mechanisms, it is necessary to clarify the effects of the interaction between pathogenic and therapeutic stimuli on intracellular signal transduction pathways.^1^ Of the many signaling pathways implicated in neuronal function, the mitogen-activated protein kinase (MAPK) and AKT-glycogen synthase kinase 3β (AKT-GSK3β) pathways have been widely investigated.^2^,^3^ These pathways are reported to play important roles in both the pathogenesis and therapeutic mechanism associated with the treatment of various psychoses, such as schizophrenia and bipolar disorders.^4^-^7^

MK-801 is a noncompetitive N-methyl-D-aspartate (NMDA) receptor antagonist that is commonly used to induce a psychosis-like state that is characterized by both negative and positive symptoms of schizophrenia. Rodents treated with MK-801 displayed psychotomimetic behavior and behaviors that correspond to the core symptoms of psychosis.^8^ Single administration of MK-801 may also result in acute behavioral effects,^9^ whereas prolonged administration of MK-801 has been shown to induce behavioral effects consistent with the cognitive deficits and negative symptoms of schizophrenia.^10^ We previously reported that MK-801 increased the phosphorylation of GSK-3β (Ser9), AKT (Ser473) and cyclic adenosine monophosphate-responsive element binding (CREB)(Ser 133) in the rat brain.^11^ We also reported that treatment with 1 mg/kg MK-801 resulted in the upregulation of phosphorylation in the mitogen-activated ERK kinase (MEK)-ERK-p90RSK pathways in the rat frontal cortex.^12^

Electroconvulsive therapy (ECT) has been proven to be very effective in managing psychotic or depressive symptoms that respond poorly to pharmacotherapy.^13^ Electroconvulsive shock (ECS), which is often used as a model for ECT in humans, has been found to increase neurogenesis in the rat hippocampus and activate cell cycle-dependent kinases in the rat frontal cortex.^14^,^15^ ECS causes massive neuronal depolarization, which triggers rapid signaling in the nervous system via synaptic transmission and axonal conductance.^16^ KCl-induced neuronal depolarization is widely used as an *in vitro* model of neuronal activity and ECS.^17^ It was previously reported that acute ECS could cause biphasic changes in the phosphorylation of GSK-3β (Ser9) in the rat brain.^18^ In addition, multiple ECS treatment was reported to affect the expression and phosphorylation of ERK1/2 in the rat frontal cortex.^19^ There were also reports that KCl-induced depolarization causes a biphasic temporal pattern of AKT (Ser473) and GSK3β (Ser9) phosphorylation in SH-SY5Y cells17 and induces neuronal proliferation.^20^

To better understand the interplay between the signaling pathways involved in the pathogenesis and therapeutic mechanisms, we tried to explore the possibility of a cellular model. Thus, we investigated the interaction between MK801, a model of psychosis, and KCl-induced depolarization, a therapeutic model of ECT in SH-SY5Y human neuroblastoma cells. The aim of this study was to analyze the interaction between MK-801 and KCl-induced depolarization and investigate its role in the regulation of AKT-GSK3β and ERK1/2 in SH-SY5Y cells. The second aim of this study was to investigate whether the interactions observed in these cell lines were reproducible in the rat frontal cortex after MK801 treatment followed by ECS.

## Methods

### Cell culture and treatments

SH-SY5Y cells were purchased from American Type Culture Collection (Manassas, VA, USA). SH-SY5Y cells were maintained in Dulbecco's modified Eagle's medium (DMEM, Sigma-Aldrich, St. Louis, MO, USA) containing 10% fetal bovine serum (FBS, JBI, Korea) and 100 U/ml penicillin/streptomycin (Sigma-Aldrich) and incubated at 37℃ in 5% CO_2_·KCl and NaCl (Sigma-Aldrich) were dissolved in DMEM. MK-801 (Tocris, Bristol, UK) was dissolved in normal saline. Before treatment, SH-SY5Y cells were washed and incubated overnight in fresh DMEM containing 10% FBS. Neuronal depolarization was induced by treating the cells with 100 mM KCl in complete medium for 5 min. The cells were then immediately washed with fresh medium and reintroduced into fresh medium. For treatment with MK-801, 1 µM MK-801 was added, and the cells were incubated for 15 min, after which the medium was replaced with fresh medium containing 1 µM MK-801. The dissociation value (K_D_) of MK-801 was 5.7 nM for the whole brain and 8.1 nM for the spinal cord.^21^ MK-801 was used at a concentration of 1 µM in order to completely block the NMDA receptor, while minimizing the direct osmotic and physically toxic effect on SH-SY5Y cells. Post-treatment, the cells were harvested after incubation in fresh medium for the indicated times.

### Animal treatments

Animals were treated in accordance with the Guide for the Care and Use of Laboratory Animals published by the National Institutes of Health (NIH). Male Sprague-Dawley rats (150-200 g) were housed for 2 weeks before the experiment and maintained under 12 h light/12 h dark conditions with food and water available ad libitum. The rats were divided into three groups of three each. The groups were given the following treatments: group 1, intraperitoneal injection of normal saline followed by sham treatment; group 2 intraperitoneal injection of normal saline followed by a single ECS; and group 3, intraperitoneal injection of 1 mg/kg MK-801, followed by a single ECS. Previous experiments have shown that AKT and GSK phosphorylation increased after treatment with MK-801 at concentrations up to 1 mg/kg in the rat frontal cortex but decreased at higher dosages.^11^ Therefore, 1 mg/kg MK-801 was selected in order to achieve maximum changes in the phosphorylation of AKT and GSK. ECS was delivered to male Sprague-Dawley rats (150-200 g) via ear-clip electrodes (130 V, 0.5 s, Medicraft B-24 apparatus). Rats were intraperitoneally injected with MK-801 or normal saline 30 min before ECS. Rats were sacrificed by decapitation at the indicated time points following each treatment, and the frontal cortices were dissected.

### Immunoblot analysis

SH-SY5Y cells were harvested with a cell scraper in lysis buffer containing 25 mM HEPES (pH 7.4), 150 mM NaCl, 0.1 mM EDTA, 1 mM Na_3_VO_4_, 1 mM NaF, 20 mM β-glycerophosphate, 1 mM phenylmethylsulfonyl fluoride, a protease inhibitor mixture (Roche Molecular Biochemicals), and 1% Triton X-100. The cells were lysed by incubating the suspension on ice for 30 min. Rat cortical tissue was homogenized in 10 v/w of icecold homogenization buffer [50 mM Tris, pH 7.4, 1% Triton X-100, 0.5% deoxycholate, 150 mM NaCl, 1 mM EGTA, 1 mM EDTA, 1 mM Na_3_VO_4_, Complete Mini Protease Inhibitor Cocktail (Roche Diagnostics, Switzerland), 1 mM DTT, 1 mM PMSF]. Homogenates were centrifuged, and supernatants were boiled with Laemmli's sample buffer. The samples were then separated by SDS-PAGE.

Antibodies against GSK3β, ERK1/2 were purchased from Santa Cruz Biotechnology (1:5000; Santa Cruz, CA, USA), and antibodies against phosphorylated GSK3β (1:1000, Ser9), phosphorylated AKT (1:1000, Ser473), phosphorylated ERK1/2 (1:1500, Thr202/Tyr204) and AKT (1:3000) were obtained from New England BioLab's Cell Signaling Group (Beverly, MA, USA). Horseradish peroxidase-conjugated goat antimouse and goat anti-rabbit IgG antibodies (1 : 3000) were obtained from Zymed (San Francisco, CA, USA). The blots were incubated with enhanced chemiluminescence substrate mix (Pierce, Rockford, IL, USA) and exposed to radiographic film (AGFA, Curix RPI, Mortsel, Belgium) in order to obtain images. The densities of the bands were quantified using an image analyzer (TINA 2.0, Raytest, Germany).

### Statistical analysis

All analyses were performed using SPSS 13.0 for Windows (SPSS Inc., Chicago, IL, USA), and the data are presented as the means±S.D. The nonparametric Mann-Whitney U test was used for non-normal data to analyze mean values, and statistical significance was established at p<0.05 (two-tailed).

## Results

### Phosphorylation of AKT at Ser473 in SH-SY5Y cells

**FIGURE 1:**
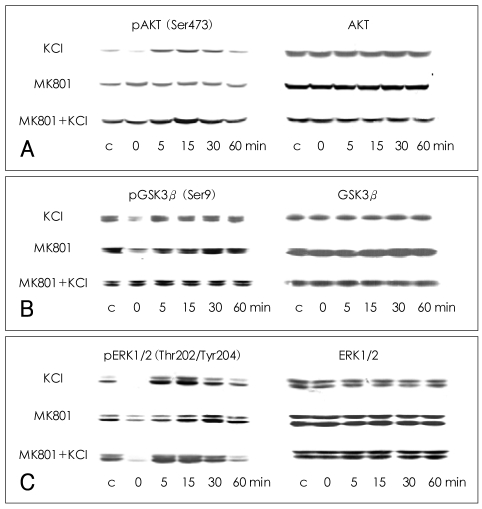
Effects of KCl-induced depolarization and MK-801 on the phosphorylation of AKT (Ser473), GSK3β (Ser9) and ERK1/2 (Thr202/Tyr204). For the interaction experiment, cells were pretreated with 1 µM MK-801 for 15 min, followed by cotreatment with 100 mM KCl for 5 min. After the KCl was washed out, MK-801 was reintroduced, and the samples were incubated for the indicated times before harvesting. Representative immunoblots of phosphorylation of (A) AKT (Ser473), (B) GSK3β (Ser9) and (C) ERK1/2 (Thr202/Tyr204) in SH-SY5Y cells harvested after exposure to 100 mM KCl-induced depolarization, 1 µM MK-801, and KCl plus MK-801 cotreatment for the indicated times are shown. C indicates the vehicle-treated controls. GSK3β: glycogen synthase kinase 3β, ERK: extracellular legulated kinase.

The phosphorylation of AKT (Ser473) was significantly decreased at 0 min after the cells were treated with 100 mM KCl for 5 min (70.3±14.1% of control value; Mann-Whitney test: t=10.00, z=-2.460, p=0.007, r=-0.87; n=4). The phosphorylation of AKT (Ser473) increased between 5 and 30 min after the initial dephosphorylation, peaked at 15 min, and then returned to baseline at 60 min. No significant changes in the phosphorylation of AKT (Ser473) were observed after treatment with MK-801 alone, though some fluctuations were observed. When the cells were treated with KCl and MK-801, the phosphorylation of AKT (Ser473) showed a pattern of change similar to that observed after treatment with KCl alone, except that no immediate dephosphorylation was observed at 0 min (94.0±14.5% of control value; n=4). There was a significant difference in phosphorylation at 0 min between the KCl-treated cells and the MK-801- and KCl-treated cells (Mann-Whitney test: t=10.00, z=-1.732, p=0.042, r=-0.61; n=4), indicating that MK-801 blocked the KCl-induced dephosphorylation of AKT (Ser473) at 0 min. The total amount of AKT did not change significantly under any of the conditions (Figure 1A).

### Phosphorylation of glycogen synthase kinase 3β at Ser9 in SH-SY5Y-cells

GSK3β (Ser9) phosphorylation was decreased at 0 min after KCl treatment, as compared with the vehicle-treated controls (74.1±26.2% of control value; Mann-Whitney test: t=10.00, z=-2.460, p=0.007, r=-0.87; n=4), and then increased slightly before returning to a basal level. The phosphorylation of GSK3β (Ser9) also decreased at 0 min after MK-801 treatment and recovered slowly to a basal level at 30-60 min. After treatment with KCl and MK-801, the phosphorylation of GSK3β (Ser9) showed a pattern of change similar to that observed after treatment with KCl alone, except that no immediate dephosphorylation was observed at 0 min (121.3±32.6% of control value). There was a significant difference in phosphorylation at 0 min between cells treated with KCl alone and those treated with MK-801 and KCl (Mann-Whitney test: t=10.00, z=-1.732, p=0.042, r=-0.61; n=4). The total amount of GSK3β did not change significantly under any of the conditions (Figure 1B).

### Phosphorylation of extracellular legulated kinase 1/2 at Thr202/Tyr204 in SH-SY5Y cells

The phosphorylation of ERK1/2 at Thr202/Tyr204 was decreased to below basal level at 0 min after treatment with KCl alone (29.3±30.0% of control value; Mann-Whitney test: t=10.00, z=-2.460, p=0.007, r=-0.87; n=4), peaked at 15 min, and then returned to baseline at 60 min. After treatment with MK-801 alone, the phosphorylation of ERK1/2 (Thr202/Tyr204) was decreased at 0 min (63.5±19.6% of control value; Mann-Whitney test: t=10.00, z=-2.460, p=0.007, r=-0.87; n=4) and then slowly recovered to basal level between 5-60 min. After cotreatment with both KCl and MK-801, the phosphorylation of ERK1/2 (Thr202/Tyr204) showed a pattern of change similar to that observed after treatment with KCl alone, exhibiting dephosphorylation at 0 min (49.0±29.6% of control value; Mann-Whitney test: t=10.00, z=-2.460, p=0.007, r=-0.87; n=4). The total amount of ERK1/2 did not change significantly under any of the conditions (Figure 1C).

### Effects of KCl and NaCl on SH-SY5Y cells

**FIGURE 2:**
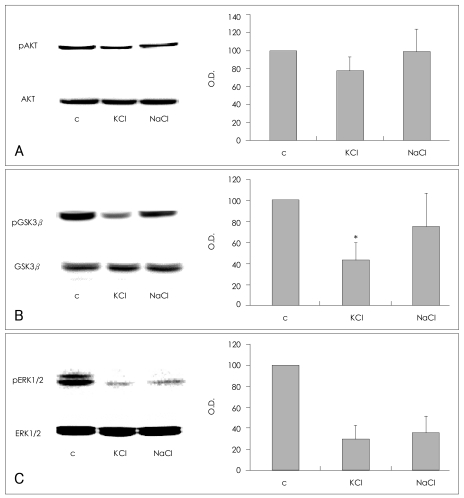
Effects of KCl and NaCl on the phosphorylation of AKT (Ser473), GSK3β (Ser9), and ERK1/2 (Thr202/Tyr204) in SH-SY5Y cells. Representative immunoblot of changes in the phosphorylation of (A) AKT (Ser473), (B) GSK3β (Ser9), and (C) ERK1/2 (Thr202/Tyr204). After SH-SY5Y cells were treated with 100 mM KCl or NaCl in complete medium for 5 min, the cells were washed and harvested for analysis. The quantitative analysis of the phosphorylation is presented in the bar graphs. The amount of phosphorylation is expressed as a percentage of the corresponding density of the vehicle-treated controls. The graphs represent the average values and standard deviations. The asterisk (*) indicates a statistically significant difference in GSK3β phosphorylation compared with the vehicle-treated controls (Mann-Whitney test: t=10.00, z=-2.460, p=0.014, r=-0.87; n=4). C indicates vehicle-treated controls. GSK3β: glycogen synthase kinase 3β, ERK: extracellular legulated kinase.

The depolarizing and osmotic effects of KCl were evaluated. SH-SY5Y cells were treated with 100 mM of KCl or NaCl for 5 min, and the effects of these treatments on the phosphorylation of AKT (Ser473), GSK3β (Ser9), and ERK1/2 (Thr202/Tyr204) were compared. The phosphorylation of AKT (Ser473) was decreased in cells treated with KCl in comparison with the vehicle-treated controls (77.6±15.3% of control value; n=4), but unchanged in cells treated with NaCl (98.8±25.2% of control value; n=4, Figure 2A). Although treatment with KCl or NaCl, both decreased GSK3β (Ser9) phosphorylation (KCl, 43.4±16.6% of control value; NaCl, 75.2±32.4% of control value; n=4, respectively), the KCl treatment was more consistent and effective than the NaCl treatment. Treatment with KCl resulted in a statistically significant decrease in GSK3β (Ser9) phosphorylation in comparison with the controls (Mann-Whitney test: t=10.00, z=-2.460, p=0.014, r=-0.87; n=4, Figure 2B). These findings indicate that both depolarization and osmosis contribute to the KCl-induced dephosphorylation of GSK3β (Ser9). KCl and NaCl each induced a similar decrease in the phosphorylation of ERK1/2 (Thr202/Tyr204)(KCl: 29.6±12.6% of control value; NaCl: 35.1±16.2% of control value; n=3, respectively), which suggests that osmotic effects may have contributed significantly to the dephosphorylation of ERK1/2 (Thr202/Tyr204) by KCl (Figure 2C).

### Phosphorylation of AKT at Ser473 in the rat frontal cortex

**FIGURE 3:**
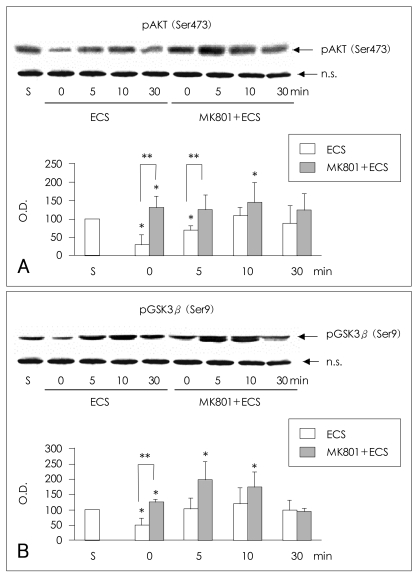
Effects of MK-801 and electroconvulsive shock on the phosphorylation of AKT (Ser473) and GSK3β (Ser9) in the rat frontal cortex. Representative immunoblots of (A) AKT (Ser473) and (B) GSK3β (Ser9) phosphorylation in the rat frontal cortex after intraperitoneal (IP) injection of saline followed by electroconvulsive shock (ECS) or after IP injection of 1 mg/kg MK-801 followed by ECS. Densitometric analysis of AKT (Ser473) phosphorylation at designated time points after saline-ECS or MK-801-ECS treatment is presented in the bar graphs. The amount of phosphorylated AKT (Ser473) or GSK3β (Ser9) is expressed as a percentage of the corresponding density of the saline-sham-treated controls (S). The data represent the average values and standard deviations of three independent experiments. A nonspecific band (n.s.) is given to show that an equal amount of protein was applied to each lane of the immunoblot. The asterisk (*) indicates statistically significant difference in phosphorylation from that of the saline-sham-treated controls (Mann-Whitney test: t=6.00, z=-2.087, p=0.019, r=-1.21, n=3). The double asterisk (**) indicates statistically significant difference in phosphorylation between the saline-ECS-treated group and the MK-801-ECS-treated group (Mann-Whitney test: t=6.00, z=-1.964, p=0.025, r=-1.14; n=3).

The phosphorylation of AKT (Ser473) was decreased at 0 and 5 min in the frontal cortex of saline-ECS-treated rats in comparison to saline-sham-treated rats (Mann-Whitney test: 0 min and 5 min respectively, t=6.00, z=-2.087, p=0.019, r=-1.21; n=3). After the initial dephosphorylation, the phosphorylation of AKT (Ser473) showed a tendency to increase and then return to baseline around 30 min in the frontal cortex of saline-ECS-treated rats. In MK-801-ECS-treated animals, the phosphorylation of AKT (Ser473) was increased at 0 min in comparison to the saline-sham-treated samples, and it remained increased until 30 minutes after ECS, with statistical significance at 0 min and 10 min (Mann-Whitney test: 0 min and 10 min respectively, t=6.00, z=-2.087, p=0.019, r=-1.21; n=3). At 0 and 5 min after ECS, the phosphorylation of AKT at Ser 473 was af-fected by pretreatment with either saline or MK-801 (Mann-Whitney test: 0 min and 5 min respectively, t= 6.00, z=-1.964, p=0.025, r=-1.14; n=3). There was no difference between the saline-ECS- and MK-801-ECS-treated groups at 10 or 30 min after ECS (Figure 3A).

### Phosphorylation of glycogen synthase kinase3β at Ser9 in the rat frontal cortex

The phosphorylation of GSK3β (Ser9) was also decreased at 0 min in the frontal cortex of saline-ECS-treated rats in comparison to saline-sham-treated rats (Mann-Whitney test: t=6.00, z=-2.087, p=0.019, r=-1.21; n=3). After the initial dephosphorylation, the phosphorylation of GSK3β (Ser9) increased and then returned to baseline after approximately 30 min in the saline-ECS-treated rat frontal cortex. In the MK-801-ECS-treated animals, the phosphorylation of GSK3β (Ser9) was increased at 0 min in comparison to the saline-sham-treated samples, remained increased until 10 min after ECS and then returned to baseline at 30 minutes after ECS (Mann-Whitney test: 0 min, 5 min and 10 min respectively t=6.00, z=-2.087, p=0.019, r=-1.21; n=3). At 0 min after ECS, phosphorylation of GSK3β (Ser9) was affected by pretreatment with either saline or MK-801 (Mann-Whitney test: 0 min, t=6.00, z=-1.964, p=0.025, r=-1.14; n=3). There was no difference between the saline-ECS-treated and MK-801-ECS-treated groups at 5,10 or 30 min after ECS (Figure 3B).

## Discussion

In this study, the interaction between MK-801 and KCl-induced depolarization and its role in the regulation of AKT-GSK3β and ERK1/2 in SH-SY5Y cells were investigated. We found that MK-801 inhibited the immediate KCl-induced dephosphorylation of AKT (Ser473) and GSK3β (Ser9) in SH-SY5Y neuroblastoma cells. MK-801 had no effect on the KCl-induced immediate dephosphorylation of ERK1/2 (Thr202/Tyr204). We also observed that ECS resulted in immediate transient dephosphorylation of AKT (Ser473) and GSK3β (Ser9), whereas pretreatment with MK-801 inhibited the immediate dephosphorylation of AKT (Ser473) and GSK3β (Ser9) produced by ECS in the rat frontal cortex. These findings suggest that the AKT-GSK3β pathway may be involved in the antagonistic interplay between MK-801 and KCl-induced depolarization/ECS.

A primary effect of KCl is to induce intracellular Ca^2+^ flow via VGCC, whereas a primary effect of MK-801 is to block the flow of intracellular Ca^2+^ via the NMDA receptor.^17^,^22^ Therefore, it is plausible that MK-801 may inhibit the KCl-induced dephosphorylation of AKT (Ser473) and GSK3β (Ser9) in a calcium-dependent manner. There have also been reports of increased phosphatase activity and reduced phosphorylation of AKT (Ser473) and GSK3β (Ser9) immediately after ECS treatment.^23^ Decreases in basal Ca^2+^ influx caused by MK-801 may inhibit the KCl-induced activation of phosphatases, which may result in the inhibition of dephosphorylation of AKT (Ser473) and GSK3β (Ser9).

The time series changes in the phosphorylation of AKT (Ser473) and GSK3β (Ser9) after treatment with MK-801 were previously reported by the authors and are therefore not shown in this study.^11^ We previously reported that the phosphorylation of AKT (Ser473) and GSK3β (Ser9) were increased in the rat frontal cortex at 15-90 minutes after treatment with MK-801.^11^ Therefore, although treatment with MK-801 alone did not result in a statistically significant increase in the phosphorylation of AKT (Ser473) or GSK3β (Ser9) in SH-SY5Y cells (Figure 1B), it is possible that an increase in the phosphorylation of AKT (Ser473) and GSK3β (Ser9) by MK-801 may have overcome the dephosphorylation induced by KCl.

Moreover, we found that, for AKT (Ser473) and GSK3β (Ser9), the interaction between ECS and MK-801 in the rat frontal cortex was similar to the interaction between KCl-induced depolarization and MK-801 in SH-SY5Y cells. These findings also suggest that the depolarizing effect of KCl may share a similar mechanism with the immediate response of ECS. In addition, the similarity in the findings in SH-SY5Y neuroblastoma cells and rat frontal cortex suggests the possibility of using SH-SY5Y as a cellular model for investigating the mechanism of interaction between MK-801 and ECS in future studies.

Our result also indicated that there is no significant interaction between MK-801 and KCl related to the dephosphorylation of ERK1/2. Furthermore, the dephosphorylation of ERK1/2 (Thr202/Tyr204) was not caused by the depolarizing effect of KCl but rather by its osmotic effect because the effect of NaCl was similar to that of KCl. Our findings show that the depolarizing effect of KCl contributes to the dephosphorylation of AKT and GSK3β and that the osmotic effect of KCl contributes to the dephosphorylation of ERK1/2 (Thr202/Tyr204). These findings are consistent with previous reports demonstrating that ECS, which is said to result in massive transient depolarization of neurons,^16^ caused an immediate decrease in the phosphorylation of AKT and GSK3β, whereas an immediate increase in the phosphorylation of ERK1/2 (Thr202/Tyr204) was observed after ECS.^23^

ERK1/2 has been found to be activated by glutamate receptor stimulation and deactivated by NMDA receptor antagonists, including MK-801, in primary cortical cultures.^24^,^25^ The results of this study also show that MK-801 treatment decreased the activation of ERK1/2 in SH-SY5Y cells. Because we performed the cell experiment in whole medium containing 5% serum in order to mimic the physiological conditions in the brain, the decrease in the activation of ERK1/2 may have resulted from blockage of the basal level of NMDA receptor activation in SH-SY5Y cells. However, we failed to observe that MK-801 blocks the initial dephosphorylation of ERK1/2 or significantly reduces the KCl-induced activation of ERK1/2 at later time points.

The roles of AKT-GSK3β and ERK1/2 in psychiatric disorders have been discussed in numerous reports on bipolar disorder and schizophrenia.^5^,^6^,^26^,^27^ Many reports have implicated that the same pathway is involved in the therapeutic mechanisms of various mood stabilizers, anti-psychotics and ECT.^28^-^31^ This study provides further evidence for the above implications by demonstrating that AKT-GSK3β and ERK1/2 are regulated by KCl-induced depolarization, a possible therapeutic model of ECS and neuronal activity, and by MK-801, a drug that induces psychotomimetic effects. The results of this study also show that an antagonistic interplay between MK-801 and KCl-induced neuronal depolarization may be involved in the regulation of AKT (Ser473) and GSK3β (Ser9). These molecular interactions may suggest a possible therapeutic mechanism of ECS in the treatment of psychosis. However, the physiological and clinical relevance of these findings need to be further elucidated.
